# Multimodal prehabilitation and perioperative immune function in patients undergoing abdominal cancer surgery

**DOI:** 10.1093/bjs/znag070

**Published:** 2026-06-06

**Authors:** Lotte M C Jacobs, Luuk D Drager, Lucas T van Eijk, Leonie S Helder, Leo A B Joosten, Dieuwke Strijker, Cornelis J H M van Laarhoven, Baukje van den Heuvel, Michiel C Warlé

**Affiliations:** Department of Surgery, Radboud University Medical Centre, Nijmegen, The Netherlands; Department of Surgery, Radboud University Medical Centre, Nijmegen, The Netherlands; Department of Anaesthesiology, Pain, and Palliative Medicine, Radboud University Medical Centre, Nijmegen, The Netherlands; Department of Internal Medicine, Radboud University Medical Centre, Nijmegen, The Netherlands; Department of Internal Medicine, Radboud University Medical Centre, Nijmegen, The Netherlands; Department of Medical Genetics, Iuliu Hatieganu University of Medicine and Pharmacy, Cluj-Napoca, Romania; Department of Surgery, Radboud University Medical Centre, Nijmegen, The Netherlands; Department of Surgery, Radboud University Medical Centre, Nijmegen, The Netherlands; Department of Surgery, Radboud University Medical Centre, Nijmegen, The Netherlands; Department of Surgery, Radboud University Medical Centre, Nijmegen, The Netherlands

## Abstract

**Background:**

Prehabilitation is an emerging preoperative strategy designed to optimize patients’ functional capacity before surgery to improve postoperative outcomes. Previous studies have demonstrated its clinical benefit, including enhanced recovery and reduced postoperative complications. However, the mechanisms underlying these benefits remain poorly understood. The aim of this exploratory study was to investigate the potential effects of prehabilitation on preoperative and postoperative immune function.

**Methods:**

This prospective study utilized data and material from a subgroup of patients participating in the F4S PREHAB trial (NL73777.091.20), which is a stepped-wedge trial investigating the effects of multimodal prehabilitation before major surgery. In this substudy, patients who underwent elective bladder, oesophageal, or rectal cancer surgery between June 2022 and November 2023 were included. Immune function was assessed at baseline, after the prehabilitation or usual care interval, and on postoperative day 1 (POD1) by examining *ex vivo* cytokine production capacity, plasma cytokine concentrations, and monocytic human leucocyte antigen DR (mHLA-DR) expression.

**Results:**

This substudy included 130 patients: 102 in the prehabilitation group and 28 in the control group. After prehabilitation, the *ex vivo* production of pro-inflammatory cytokines was reduced, whereas the *ex vivo* production of the anti-inflammatory cytokine interleukin 10 (IL-10) was increased. Plasma concentrations of interleukin 6 (IL-6) and IL-10 were lower after prehabilitation. On POD1, no significant differences in postoperative immune function were observed between the prehabilitation and control groups.

**Conclusion:**

This study suggests that multimodal prehabilitation influences basal immune function, leading to a less inflammatory state. However, as no significant differences in immune function were observed between the prehabilitation and control groups on POD1, the impact of prehabilitation on postoperative immune function may be limited.

## Teaser

This study suggests that multimodal prehabilitation influences basal immune function, leading to a less inflammatory state. However, as no significant differences in immune function were observed between prehabilitation and control groups on postoperative day 1, the impact of prehabilitation on postoperative immune function may be limited.

## Introduction

The global incidence of abdominal cancer continues to rise each year^1^. Patients with abdominal malignancies often suffer physical decline due to disease-related symptoms or side effects of neoadjuvant therapies^2^. Moreover, surgery—an integral component of curative treatment—frequently results in postoperative complications^3–6^, extending hospital stays, increasing mortality rates, and increasing healthcare costs^7^. Consequently, prehabilitation has become an increasingly common strategy in preoperative treatment that aims to optimize patients’ functional capacity before surgery, enhancing their ability to tolerate the physical stress associated with surgical interventions in order to improve surgical outcomes.

Prehabilitation uses a multimodal approach, comprising physical exercise, nutritional counselling with protein supplementation, psychological support, and aid in smoking and alcohol cessation. While previous studies reported beneficial effects of prehabilitation on outcomes such as postoperative complications and length of hospital stay, the mechanisms underlying these benefits remain poorly understood^8–10^. One mechanism to be explored involves immunology, particularly given the temporary suppression of the innate immune system during the postoperative interval, which increases the risk of infectious complications^11,12^. Surgical tissue injury triggers an inflammatory response, followed by a compensatory anti-inflammatory response to restore immune homeostasis. A stronger initial inflammatory response typically elicits a more pronounced compensatory reaction, which can lead to postoperative immunosuppression and increased vulnerability to pathogens^13^. Immunological research may offer new insights into how prehabilitation influences immune function and postoperative recovery. For instance, regular physical activity has been shown to lower plasma concentrations of pro-inflammatory cytokines, whereas low physical activity is associated with chronic low-grade inflammation^14,15^. Nutritional interventions, such as a diet rich in antioxidants like vitamins A and C, can neutralize free radicals generated during surgery^16^. Smoking disrupts both innate and adaptive immune homeostasis, by either amplifying pathogen immune responses or diminishing the immune system’s normal protective mechanisms^17^. However, the impact of multimodal prehabilitation, which integrates these components, on perioperative immune function remains largely unexplored.

The aim of this exploratory study was to assess the effects of multimodal prehabilitation on preoperative and postoperative immune function in patients undergoing elective cancer surgery.

## Methods

### Participants and study design

In the present study, data and material were derived from a subgroup of patients participating in the F4S PREHAB trial, a monocentre stepped-wedge study that evaluates the hospital-wide effects of a multimodal prehabilitation programme in patients undergoing high-impact surgery across various medical disciplines^18,19^. This immunology substudy was introduced through a predefined protocol amendment to the F4S PREHAB trial, approved before initiation of sample collection. The substudy design, endpoints, and analysis plan were specified a priori. The Institutional Review Board of Radboudumc and the local Medical Ethics Committee (METC Oost-Nederland) gave approval for the F4S PREHAB study and its later amendments including this substudy (NL73777.091.20). All participants provided written informed consent before any study-related procedures. The main study and this substudy were conducted in accordance with the principles of the Declaration of Helsinki. Results from the main study have recently been published^19^.

In this study, patients aged ≥16 years who underwent elective bladder, oesophageal, or rectal cancer surgery between June 2022 and November 2023 at Radboudumc (Nijmegen, The Netherlands) were included. Exclusion criteria of the F4S PREHAB trial comprised impaired mobility or premorbid conditions (cardiorespiratory disease) that contraindicate high-intensity exercise, cognitive disabilities, inability to read and understand the Dutch language, chronic kidney disease stage ≥3, and ASA grade ≥IV.

### Usual care and multimodal prehabilitation programme

In accordance with the stepped-wedge design of the F4S PREHAB trial, patients were allocated to either the control group (usual care) or the intervention group (prehabilitation). The control group received usual preoperative care according to Radboudumc clinical guidelines. The intervention group participated in a tailored multimodal prehabilitation programme for a duration of 3–4 weeks. This programme comprised four modalities: physical exercise, a nutritional intervention, psychological support, and aid in smoking and alcohol cessation when indicated. Exercise training was individually tailored and included both aerobic and resistance training under the supervision of a local physiotherapist. Nutritional support was guided by dietitians, and psychological and behavioural interventions were provided as needed. Further details regarding the structure and intensity of the multimodal prehabilitation programme have been described previously^18^ and are provided in the *Supplementary material*.

During the inclusion interval of the present study, the oesophageal cancer and rectal cancer clinical pathways had already transitioned to multimodal prehabilitation, whereas the bladder cancer clinical pathway included intervals of both usual preoperative care and multimodal prehabilitation.

### Study outcomes

The impact of participation in a multimodal prehabilitation programme on basal immune function was assessed for all intervention patients by measuring *ex vivo* cytokine production capacity and plasma cytokine concentrations over time. Additionally, the study evaluated the extent of postoperative immunosuppression by comparing the prehabilitation and control groups. Immunosuppression was assessed by analysing the *ex vivo* cytokine production capacity of whole blood, plasma cytokine concentrations, and monocytic human leucocyte antigen DR (mHLA-DR) expression. This comparative analysis was limited to the bladder cancer clinical pathway to ensure a homogeneous sample including both control and intervention patients.

The laboratory staff were not blinded to group allocation, as the analyses were performed by the investigators themselves.

### Sample and data collection

Baseline, tumour, and treatment characteristics and perioperative parameters were obtained from digital patient files. Data regarding functional capacity and nutritional status were gathered both at baseline and after the intervention before surgery.

The estimated maximal oxygen uptake (peakVO_2_) (ml/kg/min) was based on the Steep Ramp Test (SRT) on a cycle ergometer (Lode, Groningen, The Netherlands)^20^. An indirect one-repetition maximum (1RM) test on a leg-press machine was used to assess leg muscle strength. The handgrip strength of the dominant hand was determined with a hydraulic handgrip dynamometer. Lower limb functioning was assessed with the Five Times Sit to Stand Test (FTSST)^21^. BMI was calculated using measured body height and weight. Bioelectrical impedance analysis (Bodystat 1500) was used to determine fat-free mass.

Specifically for this study, lithium heparin (LH) and EDTA anticoagulated blood samples were drawn at three time points: at baseline, after the prehabilitation or control interval before surgery, and on postoperative day 1 (POD1). The blood tubes were kept at room temperature and processed (plasma storage and *ex vivo* assays) within 2 h of blood collection. Both the LH and EDTA tubes were centrifuged at 2970 g at room temperature for 10 min. The supernatant from the EDTA anticoagulated blood tube was centrifuged again at 16 000 g at room temperature for 10 min. Thereafter, LH and EDTA anticoagulated plasma samples were stored at −80°C until further analysis.

### *Ex vivo* cytokine production capacity

The *ex vivo* cytokine production capacity of leucocytes upon whole-blood stimulation with *Escherichia coli* lipopolysaccharide (LPS) was determined to effectively assess the functionality of the immune system. As described in previous literature^11,22^, 0.5 ml of LH anticoagulated whole blood was added to prefilled tubes with 2 ml of Dutch-modified Roswell Park Memorial Institute (RPMI) 1640 culture medium (negative control) or 2 ml of culture medium supplemented with *E. coli* LPS (serotype O55:B5; Sigma-Aldrich, St Louis, MO, USA) at a final concentration of 10 ng/ml. Culture medium (both with and without *E. coli* LPS) was supplemented with gentamicin (0.05 mg/ml), pyruvate (1 mM), and GlutaMAX (2 mM). The tubes were incubated for 24 h at 37°C with 5% CO_2_. Then, they were centrifuged at 2970 g at room temperature for 10 min and the supernatants were stored at −80°C until further analysis. Concentrations of pro-inflammatory cytokines (interleukin 6 (IL-6), interleukin 1β (IL-1β), and tumour necrosis factor (TNF)) and the anti-inflammatory cytokine interleukin 10 (IL-10) in the supernatants were measured batchwise using Human Bio-Techne R&D DuoSet ELISA according to the manufacturer’s protocol (R&D Systems, Minneapolis, MN, USA; catalogue numbers DY206, DY201, DY210, and DY217B). The plates were read using an 800TS BioTek microplate reader. Outputs are reported as absolute LPS-stimulated values.

Batch effects for *ex vivo* measurements were minimized by preparing all stimulation tubes in a single batch, analysing all samples in one measurement run, distributing samples from both study groups across plates, and including controls.

### Plasma cytokine concentrations

Concentrations of IL-6, TNF, and IL-10 in EDTA anticoagulated plasma were measured batchwise using a simultaneous Luminex assay (Milliplex; Millipore, Billerica, MA, USA) according to the manufacturer’s instructions. Batch effects were minimized by distributing samples from both study groups across plates, analysing all samples in a single run, and including manufacturer-recommended controls.

### Flow cytometric quantification of mHLA-DR expression

Blood processing for flow cytometric analysis was initiated within 1 h after blood collection when samples were stored at room temperature or within 4 h after blood collection when samples when stored in the refrigerator. For all samples of patients who underwent cystectomy, 50 µl of EDTA anticoagulated blood was stained with 20 µl of QuantiBrite HLA-DR/Monocyte reagent (QuantiBrite anti-HLA-DR (PE)/anti-Monocyte (CD14) PerCP-Cy5.5; Becton Dickinson, San Jose, CA, USA) at room temperature in the dark for 25 min. The stained whole blood, along with 50 µl of unstained EDTA anticoagulated blood (control), was lysed using FACS lysing solution (Becton Dickinson) for 10 min. The cells were then measured using a CytoFLEX flow cytometer (Beckman Coulter). Batch effects were minimized through standardization procedures alongside routine start-up quality control.

Data analysis was performed using Kaluza Analysis software (version 2.2.0). Monocytes were identified based on CD14 expression, and mHLA-DR expression was then measured on their surface using a mono-parametric histogram. This was reported as the geometric mean of fluorescence intensity related to the entire monocyte population. These results were converted into the number of antibodies bound per cell using calibrated PE beads (BD QuantiBrite™ PE Beads; Becton Dickinson).

### Statistical analyses

Baseline characteristics and data regarding prehabilitation outcomes are described for all intervention patients, and separately for patients in the control and intervention groups who underwent bladder cancer surgery. Continuous data were analysed using independent-samples *t* tests or Mann–Whitney *U* tests for normally and non-normally distributed data respectively. Categorical data were analysed using chi-squared and Fisher’s exact tests. Friedman tests with Dunn’s post-hoc test were performed to determine differences between the different time points within the total prehabilitation group. Due to the exploratory nature of this study, correction for multiple testing was not applied. The Mann–Whitney *U* test was used to determine differences in *ex vivo* cytokine production, plasma cytokine concentrations, and mHLA-DR expression between the control and prehabilitation groups in the cystectomy cohort. Correlations between physical capacity outcomes and immune parameters were determined using Spearman’s rank correlation (Spearman’s *r*).

Plasma cytokine concentrations below the detection limit as determined by Luminex assay were considered equal to the lowest detectable concentrations. Statistical analyses were performed using SPSS^®^ (IBM, Armonk, NY, USA; version 29) and figures were created using GraphPad Prism version 9 (GraphPad Software, Boston, MA, USA). *P* < 0.050 was considered statistically significant.

## Results

**Figure 1:**
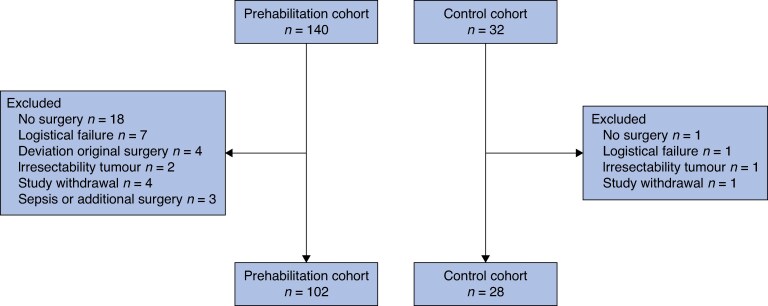
Flow chart of patient inclusion

Between June 2022 and November 2023, 172 patients were enrolled in this substudy. During this interval, 140 patients who underwent bladder, oesophageal, or rectal cancer surgery were allocated to the prehabilitation cohort, of whom 38 were excluded for various reasons (*Fig. 1*). In the control group, 32 patients who underwent bladder cancer surgery were included. Exclusion of 4 patients resulted in a final control cohort of 28 patients (*Fig. 1*).

### Baseline descriptives

The prehabilitation group had a mean(s.d.) age of 67.9(9.5) years and 74.5% of the patients were male (*Table 1*). The prehabilitation cohort comprised 30 bladder cancer patients, 44 oesophageal cancer patients, and 30 rectal cancer patients. Patients in the prehabilitation cohort participated in a median of 7 (interquartile range (i.q.r.) 5–10) exercise sessions. Patients in the bladder cancer cohort had a mean(s.d.) age of 72.5(8.2) and 68.7(10.8) years in the prehabilitation group and the control group respectively and were predominantly male. There were no significant differences in baseline characteristics—including age, sex, smoking status, ASA grade, and neoadjuvant treatment—between the prehabilitation and control groups for patients who underwent bladder cancer surgery.

**Table 1:** Baseline characteristics

	All prehabilitation patients (*n* = 102)	Bladder cancer patients (*n* = 58)		
		Prehabilitation (*n* = 30)	Control (*n* = 28)	*P*
Age (years), mean(s.d.)	67.9(9.5)	72.5(8.2)	68.7(10.8)	0.137
**Sex**				0.230
Male	76 (74.5)	27 (90.0)	22 (78.6)	
Female	26 (25.5)	3 (10.0)	6 (21.4)	
**Smoking status**				0.515
Current	10 (9.8)	5 (16.7)	6 (22.2)	
Former	63 (61.8)	20 (66.7)	14 (51.9)	
Never	29 (28.4)	5 (16.7)	7 (25.9)	
**ASA grade**				0.293
1–2	73 (71.6)	17 (56.7)	12 (42.9)	
3	29 (28.4)	13 (43.3)	16 (57.1)	
**Tumour location**				NA
Bladder	30 (29.4)	30 (100)	28 (100)	
Oesophagus	44 (43.1)	0 (0)	0 (0)	
Rectum	28 (27.5)	0 (0)	0 (0)	
**Neoadjuvant treatment**				0.394
None	36 (35.3)	22 (73.3)	18 (64.3)	
Chemotherapy	12 (11.8)	7 (23.3)	10 (35.7)	
Radiotherapy	7 (6.9)	1 (3.3)	0 (0)	
Chemoradiotherapy	47 (46.1)	0 (0)	0 (0)	
Exercise sessions, median (i.q.r.)	7 (5–10)	6.5 (5–9)	NA	NA

Values are *n* (%) unless otherwise indicated. NA, not applicable; i.q.r., interquartile range.

### Functional capacity and nutritional parameters

The prehabilitation cohort improved with regard to estimated peakVO_2_ and the 1RM leg-press test before surgery, with additional increments in BMI and fat-free mass (*Table 2*). In the bladder cancer prehabilitation group, there were significant improvements for estimated peakVO_2_ (mean(s.d.) of 22.4(3.8) *versus* 23.1(3.6) ml/kg/min, *P* = 0.023) and the 1RM leg-press test (mean(s.d.) of 94.5(34.0) *versus* 113.6(46.9) kg, *P* = 0.007) between baseline and before surgery.

**Table 2:** Changes in functional capacity and nutritional status

	Baseline	Before surgery	*P*
**Estimated peakVO_2_ (ml/kg/min)**			
Prehabilitation cohort	23.1(3.9)	23.6(3.8)	0.002*
Bladder cancer control	21.6(3.5)	21.9(3.8)	0.207
Bladder cancer intervention	22.4(3.8)	23.1(3.6)	0.023*
**1RM leg-press test (kg)**			
Prehabilitation cohort	106.5(46.3)	125.5(49.0)	<0.001*
Bladder cancer control	108.7(41.2)	121.7(32.9)	0.121
Bladder cancer intervention	94.5(34.0)	113.6(46.9)	0.007*
**Handgrip strength (kg)**			
Prehabilitation cohort	37.2(9.6)	38.2(9.2)	0.086
Bladder cancer control	41.0(10.5)	36.9(14.4)	0.143
Bladder cancer intervention	39.7(8.0)	38.6(9.2)	0.292
**FTSST (s)**			
Prehabilitation cohort	9.6(3.5)	8.8(2.8)	0.004*
Bladder cancer control	9.5(2.8)	9.2(2.8)	0.657
Bladder cancer intervention	10.9(5.0)	9.5(3.9)	0.078
**BMI (kg/m^2^)**			
Prehabilitation cohort	26.0(4.1)	26.5(4.2)	<0.001*
Bladder cancer control	28.4(3.4)	28.5(3.6)	0.685
Bladder cancer intervention	26.6(4.1)	26.8(4.3)	0.199
**Fat-free mass (kg)**			
Prehabilitation cohort	57.0(10.0)	57.9(9.8)	0.008*
Bladder cancer control	60.2(8.6)	58.2(6.6)	0.100
Bladder cancer intervention	59.2(9.5)	59.7(9.3)	0.277

Values are mean(s.d.). *Statistically significant. peakVO_2_, maximal oxygen uptake; 1RM, one-repetition maximum; FTSST, Five Times Sit to Stand Test.

Parameters regarding functional capacity and nutritional status were not significantly different between baseline and before surgery in the bladder cancer control group.

### Effect of prehabilitation on basal immune function

**Figure 2:**
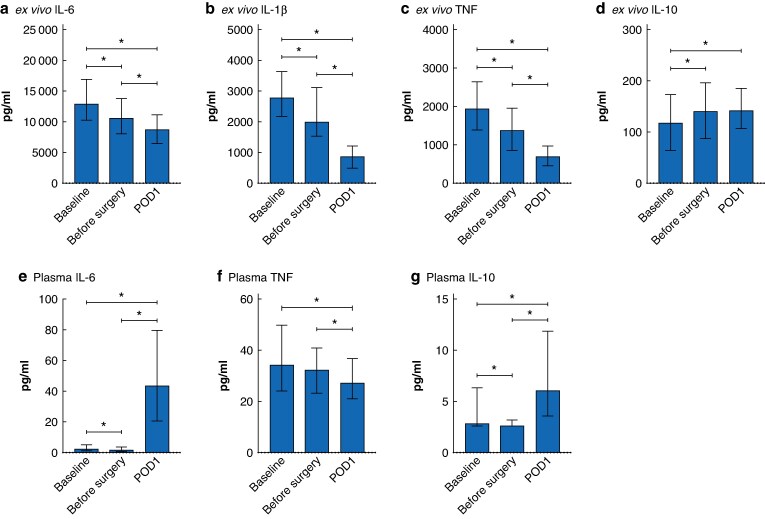
**Perioperative immune function following prehabilitation. a–d** Cytokine concentrations in supernatants after 24 h of whole-blood stimulation with *Escherichia coli* LPS and **e–g** plasma cytokine concentrations at baseline, before surgery, and on POD1 for patients who underwent cancer surgery after an interval of multimodal prehabilitation (*n* = 102). Values are median (i.q.r.). *Significant difference between time points (*P* < 0.050). LPS, lipopolysaccharide; POD1, postoperative day 1; i.q.r., interquartile range; IL-6, interleukin 6; IL-1β, interleukin 1β; TNF, tumour necrosis factor; IL-10, interleukin 10.

For patients who underwent multimodal prehabilitation, the *ex vivo* production of all three pro-inflammatory cytokines (IL-6, IL-1β, and TNF) was significantly reduced after the prehabilitation interval compared with baseline (median (i.q.r.): IL-6 = 10 551 (9122–11 745) *versus* 12 882 (11 569–14 144) pg/ml, *P* < 0.001; IL-1β = 1988 (1755–2347) *versus* 2781 (2432–2995) pg/ml, *P* < 0.001; and TNF = 1370 (1111–1514) *versus* 1935 (1663–2170) pg/ml, *P* < 0.001) and the *ex vivo* production of the anti-inflammatory cytokine IL-10 was increased (median (i.q.r.): 140 (117–164) *versus* 118 (90–136) pg/ml, *P* = 0.009) (*Fig. 2*). Additionally, plasma concentrations of IL-6 and IL-10 were significantly lower after the prehabilitation interval compared with baseline (median (i.q.r.): IL-6 = 1.5 (1.1–2.2) *versus* 2.2 (1.7–3.2) pg/ml, *P* = 0.025; and IL-10 = 2.6 (2.6–2.6) *versus* 2.8 (2.6–3.6) pg/ml, *P* = 0.006).

### Postoperative immunosuppression in bladder cancer patients

**Figure 3:**
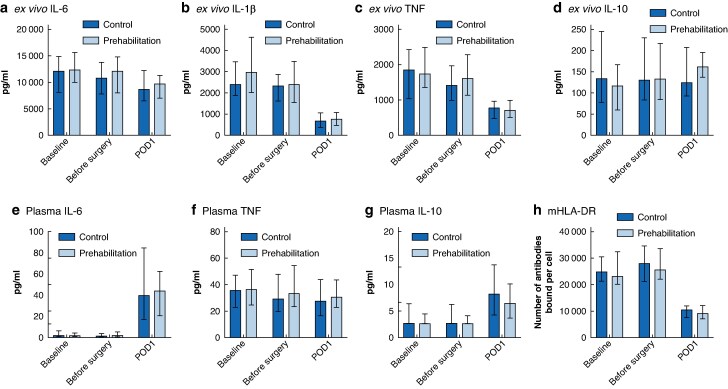
**Perioperative immune function after prehabilition versus control. a–d** Cytokine concentrations in supernatants after 24 h of whole-blood stimulation with *Escherichia coli* LPS, **e–g** plasma cytokine concentrations, and **h** mHLA-DR expression at baseline, before surgery, and on POD1 for patients who underwent bladder cancer surgery (*n* = 28 for the control group and *n* = 30 for the intervention group). Values are median (i.q.r.). No significant differences were found between control and intervention bladder cancer patients. LPS, lipopolysaccharide; mHLA-DR, monocytic human leucocyte antigen DR; POD1, postoperative day 1; i.q.r., interquartile range; IL-6, interleukin 6; IL-1β, interleukin 1β; TNF, tumour necrosis factor; IL-10, interleukin 10.

*Ex vivo* cytokine production of IL-6, IL-1β, TNF, and IL-10 did not differ between the prehabilitation and control groups on POD1. Similarly, the concentrations of IL-6, TNF, and IL-10 in plasma, as well as mHLA-DR expression, were comparable between the control and intervention groups across all time points (*Fig. 3*). Perioperative leucocyte and monocyte counts varied over time, with peak levels observed on POD1 (*Table S1*). Cell counts were similar between the groups across all time points.

### Correlations between functional capacity and postoperative immune function

The difference in estimated peakVO_2_ during the prehabilitation interval (Δ estimated peakVO_2_) negatively correlated with *ex vivo* cytokine production of IL-6 on POD1 (*r* = −0.257, *P* = 0.020). The 1RM leg-press test at the end of prehabilitation correlated with *ex vivo* cytokine production of IL-1β on POD1 (*r* = 0.335, *P* = 0.002). No correlations were found between functional capacity parameters and plasma cytokine concentrations or mHLA-DR expression.

## Discussion

This exploratory study indicates that multimodal prehabilitation influenced basal immune function, reducing the *ex vivo* production of pro-inflammatory cytokines and increasing the production of the anti-inflammatory cytokine IL-10 by the end of the prehabilitation interval. Additionally, plasma concentrations of IL-6 and IL-10 were lower after prehabilitation. However, despite these preoperative effects, no significant differences in postoperative immune function were observed between control and prehabilitation groups for patients who underwent bladder cancer surgery.

The influence of multimodal prehabilitation on perioperative immune function has not been extensively studied. To the authors’ knowledge, only one study has investigated this relationship, focusing on frail elderly patients who underwent elective gastric cancer surgery^23^. That study reported significant reductions in plasma levels of IL-6, IL-10, IL-1β, and TNF after prehabilitation, which aligns with the findings of the present study, where IL-6 and IL-10 concentrations were also reduced after prehabilitation. Furthermore, improved physical capacity observed in both studies is consistent with the broader literature showing that regular physical activity is linked to a reduction in basal pro-inflammatory cytokines^15,24^. Collectively, these findings suggest a potential association between multimodal prehabilitation and reduction of chronic low-grade inflammation.

An important aspect of this study was the assessment of *ex vivo* cytokine production capacity upon whole-blood stimulation with LPS, which has not previously been explored during prehabilitation. Assessing *ex vivo* cytokine production capacity, particularly with regard to TNF, is a widely used approach to quantify innate immune function, with reduced production capacity linked to poor outcomes in critical illness^25,26^. In the present study, it was found that prehabilitation reduced the production of pro-inflammatory cytokines and increased the production of the anti-inflammatory cytokine IL-10. This suggests a shift towards a less inflammatory state, which could indicate that immune cells are in a more quiescent or less activated state after prehabilitation. However, no differences in *ex vivo* cytokine production were observed on POD1 between the prehabilitation and control groups for the bladder cancer cohort. This may be attributed to the immunosuppressive effects of surgery, which potentially dominate over the effects of prehabilitation or other perioperative interventions^27,28^. Research has demonstrated that the impact of surgical trauma on immune function generally outweighs the influence of factors such as anaesthesia^29^. Additionally, despite improvements in functional capacity and body composition in the main study^30^, hospital-wide implementation of multimodal prehabilitation did not reduce postoperative complications or length of hospital stay^19^. Together, these findings suggest that any effects of prehabilitation on postoperative immune function are likely overshadowed by the profound impact of surgical trauma.

This study hypothesized that prehabilitation would positively influence perioperative immune homeostasis, a crucial factor in cancer patients. Inflammation is frequently associated with cancer development and progression. Tumour-extrinsic inflammation, driven by factors like obesity, smoking, and alcohol consumption, has been shown to increase cancer risk^31^. Within the tumour microenvironment, persistent chronic inflammation contributes to tumour development, progression, and metastasis^23,31^. Multiple anti-inflammatory agents have been proposed for cancer prevention and treatment, predominantly as adjuvants for conventional therapies^32^. However, their use is often limited due to undesirable side effects. Therefore, prehabilitation could serve as a complementary strategy to help reduce chronic inflammation, potentially improving immune regulation and overall patient outcomes.

While prehabilitation had a beneficial effect on preoperative immune function in this study, this did not translate into significant differences in postoperative immune function between the control and prehabilitation groups. This discrepancy is likely due to the profound immune changes induced by surgery, which may have overshadowed the effects of prehabilitation. Assessing immune function on POD1 might have therefore been too early to capture meaningful differences, as the immediate effects of surgical stress are likely at their peak. Evaluating immune responses at later time points, such as postoperative day 3, postoperative day 7, and postoperative day 30, could offer better insight into whether prehabilitation influences recovery of immune function in the postoperative phase. Furthermore, the modest improvements in physical function observed in this study—while statistically significant—were relatively small, which may limit their potential impact on immune function. More substantial improvements in physical capacity before surgery might lead to more pronounced changes in immune cell function, although the lack of clear correlations between changes in physical capacity (for example estimated peakVO_2_ and the 1RM leg-press test) and immune parameters in the present study makes this hypothesis speculative.

Several limitations of this study must be acknowledged. Due to the stepped-wedge design of the F4S PREHAB trial, the control group was relatively small and included only bladder cancer patients. This limited the ability to directly compare the bladder cancer cohort with the oesophageal and rectal cancer cohorts and reduced the statistical power to detect differences between the control and prehabilitation groups. Furthermore, factors such as age, sex, smoking status, and stress may have influenced immune function during both the control and prehabilitation intervals, though adjustments for these potential confounders were not feasible in this exploratory study. Lastly, patients in the control group were informed about the concept of prehabilitation and its potential benefits, which could have influenced their behaviour. Nevertheless, significant improvements in physical functioning parameters were observed only in the prehabilitation groups.

In conclusion, this study suggests that multimodal prehabilitation modulates basal immune function, resulting in a less inflammatory state. However, no significant differences in postoperative immune function were observed between the control and prehabilitation groups on POD1, suggesting that the impact of prehabilitation on early postoperative immune function may be limited. As the POD1 time point may have been too early to detect meaningful differences in postoperative immunocompetence, and immune recovery extends beyond this early phase, investigating later time points is likely to be more informative for recovery trajectories. Further research involving larger cohorts, more substantial changes in physical conditioning, and later immune time points is needed to clarify the clinical relevance of prehabilitation in modulating immune function and enhancing perioperative outcomes.

## Supplementary Material

znag070_Supplementary_Data

## Data Availability

The data sets generated and analysed for this study are not publicly available. Data will be made available upon reasonable request. Requests for access to the data from this pooled-data analysis can be submitted via e-mail to lotte.jacobs@radboudumc.nl.
