# Supplementary material for: Multimodal prehabilitation and perioperative immune function in patients undergoing abdominal cancer surgery
Source: Br J Surg. 2026 Jun 6;113(7):znag070. doi: 10.1093/bjs/znag070 (PMC13367574; doi:10.1093/bjs/znag070)
Supplement: znag070_Supplementary_Data [file znag070_supplementary_data.docx]

**The Role of Multimodal Prehabilitation in Mitigating Surgery-Induced Immunosuppression following Abdominal Cancer Surgery**

Lotte M.C. Jacobs^1*^, Luuk D. Drager^1*^, Lucas T. van Eijk^2^, Leonie S. Helder^3^, Leo A.B. Joosten^3,4^, Dieuwke Strijker^1^, Cornelis J.H.M. van Laarhoven^1^, Baukje van den Heuvel^1^, Michiel C. Warlé^1^

**Affiliations**

1. Department of Surgery, Radboud University Medical Center, Nijmegen, The Netherlands
2. Department of Anaesthesiology, Pain and Palliative medicine, Radboud University Medical Center, Nijmegen, The Netherlands
3. Department of Internal Medicine, Radboud University Medical Center, Nijmegen, The Netherlands
4. Department of Medical Genetics, Iuliu Hatieganu University of Medicine and Pharmacy, Cluj-Napoca, Romania

*Authors share first authorship

**Corresponding author:**

Lotte M.C. Jacobs

[Lotte.jacobs@radboudumc.nl](mailto:Lotte.jacobs@radboudumc.nl)

Geert Grooteplein Zuid 10, 6525 GA Nijmegen, the Netherlands

**Supplementary Materials - Index**

| **Supplementary Appendices** |  |
| --- | --- |
| Details regarding the multimodal prehabilitation program | *page 3* |
| Supplementary Table 1 | *Page 5* |
| References | *page 6* |
|  |  |
|  |  |

**Supplementary Methods**

**Details regarding the multimodal prehabilitation program**

The F4S PREHAB intervention contained a tailored multimodal prehabilitation program for a minimum of three weeks. The program comprised a physical exercise program, nutritional support, psychological support, a smoking cessation program, and alcohol cessation advice. The program has been described in detail previously ^1^. The description below is reproduced to ensure completeness and to address the requirements regarding the operational definition of prehabilitation.

The exercise program consisted of three 60-minute sessions per week supervised by a local physiotherapist close to a patient’s home, and included both high-intensity interval training (HIIT) on a cycle ergometer and resistance exercise training. The HIIT component started with a 2-minute warm-up followed by alternating intervals of four minutes at high intensity (at 90% of the maximum short exercise capacity (MSEC), estimated by the Steep Ramp Test) and three minutes at light intensity (at 30% of MSEC). The MSEC was estimated by the Steep Ramp Test at baseline ^2^. The SRT started after a 3-minute warming-up process without resistance. During the test, the workload increased by 25W every 10 seconds, starting at 25W. The test ended when the cycling pedal frequency fell below 60 rpm. The MSEC was calculated as the workload of the last completed stage plus 2.5W for each second in the current stage ^3^. Absolute workload was increased when participants reported a Borg rating of perceived exertion score below 15 or demonstrated a heart rate <85% of their estimated maximum during the high-intensity intervals. The highest recorded heart rate observed during the baseline Steep Ramp Test was used as the participant’s estimated maximal heart rate for guidance during training sessions. The workload was reduced when participants reported a Borg rating of perceived exertion score of 18 or higher. Resistance training targeted major muscle groups through six exercises: leg press, chest press, abdominal crunch, low row, lat pulldown, and step up. Patients performed two sets of 10 repetitions of machine-based exercises. The initial training load was set at 65% of the estimated one-repetition maximum (1RM), with a progressive increase of 5% per week. To ensure patient safety ^4^, 1RM was derived from an indirect 1 repetition maximum protocol ^5^. In case patients were treated with neoadjuvant treatment, functional tests at baseline were performed ≥14 days after the last administration to minimize acute treatment effects. On non-supervised days, patients were advised to engage in 60 minutes of moderate-intensity aerobic exercise.

Nutritional support was provided by a registered in-hospital dietitian, who offered individualized dietary guidance to optimize energy, micronutrient intake, and a daily protein intake of at least 1.5 g/kg bodyweight. Each patient received a baseline consultation with a dietitian that included screening for malnutrition (e.g., weight loss, BMI, and dietary intake), with additional sessions provided as needed. To support nutritional goals, patients were supplied with high-quality whey protein shakes (containing 30 g of whey protein and 20 µg vitamin D) and daily multivitamin supplements covering 50% of the recommended daily intake. Patients were advised to consume the protein shakes daily and directly after each supervised training session.

Psychological support was offered when indicated based on Hospital Anxiety and Depressions Scale scores ≥15. This cut-off has been shown to provide high sensitivity and specificity for detecting syndromal depression in patients with cancer ^6^. Referrals were made to clinical psychologists, who provided individualized counseling sessions focusing on anxiety reduction, stress management, and coping strategies tailored to the upcoming surgical procedure (e.g., relaxation techniques, cognitive restructuring, and practical preparation guidance). Finally, patients who were active smokers were provided access to a comprehensive smoking cessation program (SineFuma), and all patients received instructions to abstain from alcohol consumption during the preoperative period.

**Supplementary Table 1**

| **Cell counts – bladder cancer patients** | | | | | | |
| --- | --- | --- | --- | --- | --- | --- |
|  | **Prehabilitation patients (n = 30)** | | | **Control patients (n = 28)** | | |
| *10^3 / mL | **Baseline** | **Preoperative** | **POD1** | **Baseline** | **Preoperative** | **POD1** |
| **Leukocyte count, median (IQR)** | 7.3 (6.5 – 9.3) | 7.5 (6.1 – 9.2) | 12.7 (10.6 – 15.5) | 6.8 (4.8 – 8.8) | 6.8 (6.1 – 8.4) | 10.9 (9.5 – 14.8) |
| **Monocyte count, median (IQR)** | 0.65 (0.51 – 0.75) | 0.55 (0.47 – 0.78) | 1.09 (0.95 – 1.36) | 0.59 (0.50 – 0.70) | 0.63 (0.51 – 0.74) | 1.01 (0.79 – 1.21) |
| **Abbreviations:** IQR: interquartile range | | | | | | |

**Supplementary References**

1. Drager LD, van den Heuvel B, Strijker D, van Laarhoven C, Buffart LM, Verlaan S. Changes in Functional Capacity and Body Composition After a Multimodal Prehabilitation Program in Patients with Cancer undergoing Abdominal Surgery. *Ann Surg Oncol* 2026.

2. Stuiver MM, Kampshoff CS, Persoon S, Groen W, van Mechelen W, Chinapaw MJM, Brug J, Nollet F, Kersten MJ, Schep G, Buffart LM. Validation and Refinement of Prediction Models to Estimate Exercise Capacity in Cancer Survivors Using the Steep Ramp Test. *Arch Phys Med Rehabil* 2017;**98**(11): 2167-2173.

3. Meyer K, Samek L, Schwaibold M, Westbrook S, Hajric R, Beneke R, Lehmann M, Roskamm H. Interval training in patients with severe chronic heart failure: analysis and recommendations for exercise procedures. *Med Sci Sports Exerc* 1997;**29**(3): 306-312.

4. Rosenberger F, Schneider J, Schlueter K, Paratte JL, Wiskemann J. Vertebral fracture during one repetition maximum testing in a breast cancer survivor: A case report. *Medicine (Baltimore)* 2021;**100**(20): e25705.

5. Brzycki M. Strength Testing—Predicting a One-Rep Max from Reps-to-Fatigue. *Journal of Physical Education, Recreation & Dance* 1993;**64**(1): 88-90.

6. Vodermaier A, Millman RD. Accuracy of the Hospital Anxiety and Depression Scale as a screening tool in cancer patients: a systematic review and meta-analysis. *Support Care Cancer* 2011;**19**(12): 1899-1908.
